# Effects of movement representation techniques on motor learning of thumb-opposition tasks

**DOI:** 10.1038/s41598-020-67905-7

**Published:** 2020-07-23

**Authors:** Ferran Cuenca-Martínez, Luis Suso-Martí, Jose Vicente León-Hernández, Roy La Touche

**Affiliations:** 1 Departmento de Fisioterapia, Centro Superior de Estudios Universitarios CSEU La Salle, Universidad Autónoma de Madrid, Madrid, Spain; 2 Motion in Brains Research Group, Institute of Neuroscience and Sciences of the Movement (INCIMOV), Centro Superior de Estudios Universitarios CSEU La Salle, Universidad Autónoma de Madrid, Madrid, Spain; 3 Instituto de Neurociencia y Dolor Craniofacial (INDCRAN), Madrid, Spain; 4 Instituto de Investigación Sanitaria del Hospital Universitario La Paz (IdiPAZ), Madrid, Spain; 5 Department of Physiotherapy, Universidad Cardenal Herrera-CEU, CEU Universities, Valencia, Spain

**Keywords:** Skeletal muscle, Brain

## Abstract

The present work is the first study that assess long run change after motor learning. The study’s main objective was to evaluate the short to medium-term impact of motor imagery (MI) and action observation (AO) on motor learning of a sequence of thumb-opposition tasks of increasing complexity. We randomly assigned 45 participants to an AO, MI, or placebo observation (PO) group. A sequence of 12 thumb-opposition tasks was taught for 3 consecutive days (4 per day). The primary outcome was accuracy. The secondary outcomes were required time and perfect positioning. The outcomes were assessed immediately after the intervention and at 1 week, 1 month and 4 months postintervention. Regarding the primary outcome, AO group had significantly higher accuracy than the MI or PO group until at least 4 months (*p* < 0.01, *d* > 0.80). However, in the bimanual positions, AO was not superior to MI at 1 week postintervention. Regarding secondary outcomes, AO group required less time than the MI group to remember and perform the left-hand and both-hand gestures, with a large effect size (*p* < 0.01, *d* > 0.80). In terms of percentage of perfect positions, AO group achieved significantly better results than the MI group until at least 4 months after the intervention in the unimanual gestures (*p* < 0.01, *d* > 0.80) and up to 1 month postintervention in the bimanual gestures (*p* = 0.012, *d* = 1.29). AO training resulted in greater and longer term motor learning than MI and placebo intervention. If the goal is to learn some motor skills for whatever reason (e.g., following surgery or immobilization.), AO training should be considered clinically.

## Introduction

The motor learning of complex tasks involves a series of closely linked neurophysiological processes, which include motor output, somatosensory afferences, and central processing to establish certain movement parameters (e.g., strength, speed, and direction)^1^.

Mental practice applied to motor learning has been widely studied in the field of cognitive neurosciences and sports psychology. The "functional equivalence" hypothesis^1^ proposes that mental simulation processes share certain cerebral representations along with processes of preparation and real motor execution^3^. Neuroimaging studies have revealed that, during mental practice, there is neurophysiological activation of the brain areas involved in the planning and execution of voluntary movement (primary motor cortex, supplementary motor area, cerebellum, premotor area, the inferior and superior parietal lobule and the basal ganglia) similar to the processes when the movement is actually performed^4,5^.

Two well-known methods for motor skill learning are action observation (AO) and motor imagery (MI), the latter of which is defined as a dynamic mental process that involves the internal representation of an action without its actual motor output^6^. MI has been shown to facilitate the motor learning of various skills in certain contexts and settings such as golf^7^ tennis^8^ trampoline routines^9^, music^10^ dance^11^ and even surgical skills^12^.

AO, however, evokes an internal, real-time simulation of the actions being observed^13^ AO provokes an automatic activation in the observer of the same cerebral areas related to the planning and actual execution of the observed action^14^. Previous studies have shown that AO can lead to the motor learning of gestures observed from the visual information acquired, even without their actual execution^15^.

Growing scientific interest in the role of mental practice in motor learning has led to further studies to clarify the differences between AO and MI in improving motor performance. Although both processes appear to be effective, previous studies have shown that AO appears to be more effective than MI, at least in the rapid early phase of motor learning^16,17^. However, there is still a lack of scientific literature regarding the potential of these techniques and whether they can, in isolation, consolidate the learning of new motor gestures in the short/medium term with a minimal training. The potential for minimal intervention over time has not yet been investigated. This may offer interesting data when it comes to guiding the dosage of mental practice, an aspect with a lack of consensus so far. It is important to stress that MI has a relevant distinctive feature. Subjects can create changing scenes and diverse situations through MI. It is therefore that the main advantage of MI compared with AO is that scenarios can be changed and adapted to the subject's context.

One of the most studied body regions in the field of mental practice are the hands, due to their high functionality and significance^18–20^. To our knowledge, however, there have been no research studies that have evaluated motor learning through thumb-opposition specific tasks. The thumb is frequently used in daily life activities and leading to a learning process in this body region could have a significant impact on people's lives. Clinically, improved neuro-sensorimotor control, leading to a process of motor relearning after prolonged disuse and improving certain peripheral physical variables, such as strength and active range of motion, could be key aspects for reducing disability and increasing functionality^21, 22^. Another key aspect that we think will be able to influence the motor learning process through movement representation techniques is the ability to imagine movements. It has been argued that the ability to imagine is an important factor when performing mental practice^22^. For example, Martin et al.^23^ have suggested that an individual’s ability to imagine movements can determine the effectiveness of its use. The authors hypothesize that good imager is expected to show greater benefits resulting from practice.

Therefore, the primary objective of the present study was to evaluate the short to medium-term impact of MI and AO in isolation on the motor learning of a sequence of manual motor positions of increasing complexity in terms of accuracy compared with a placebo intervention. The secondary objectives were to evaluate the required time and the percentage of perfect positions that resulted from these mental practice interventions compared with a placebo intervention (also in the short to medium term). In addition, we also aimed to assess the effects on motor learning based on the ability to imagine movements in order to verify whether good imagers showed greater benefits than poor imagers accordingly to each intervention.

## Methods

### Study design

We conducted a single-blind randomized controlled study whose protocol followed the Consolidated Standards of Reporting Trials (CONSORT) statement on randomized trials of nonpharmacological treatments^23^.

### Participant recruitment

A total of 45 asymptomatic volunteers were recruited between October 2018 and June 2019 from the local community through social media and e-mail. The inclusion criteria were as follows: (a) no symptoms and (b) age 18–65 years. The exclusion criteria were the following: (a) any knowledge of physical therapy or movement representation techniques; (b) age younger than 18 years; (c) pain at the time of the study; and (d) any type of neurological disease. All data were collected at the La Salle University Center for Advanced Studies.

### Randomization

Randomization was performed using a computer-generated random sequence table with a balanced 3-block design (GraphPad Software, Inc., CA, USA). An independent researcher generated the randomization list, and a research team member who was not involved in the assessment or intervention of the participants was in charge of the randomization and maintained the list. Those included were randomly assigned to one of the three groups using the random sequence list, ensuring concealed allocation.

### Blinding

The assessments and interventions were performed by different researchers. The evaluator was blinded to the participant's assignment when performed the measurements and recorded the data. The participants were asked not to make any comments to the researcher performing the measurements. It is therefore that the evaluator did not know at any time what intervention each participant had received at the time of the results assessment.

### Interventions

#### Motor imagery

All participants in the MI group were informed of the procedure at the beginning of the intervention and underwent a familiarization session regarding the intervention they were going to perform. The participants were asked to memorize the following numbering system for the fingers of each hand:

- For the left hand: number 2 for the second finger (index finger), number 3 for the third finger, number 4 for the fourth finger and number 5 for the fifth finger.
- For the right hand: number 6 for the fifth finger, number 7 for the fourth finger, number 8 for the third finger and number 9 for the second finger (Supplementary Appendix 1).

This method of attributing numbers to fingers to perform motor imagination tasks is similar to that done by Debarnot et al.^24^ This familiarization and memorization session was separate from the training sessions and did not include any positions that were to be evaluated later.

After the participants had memorized this step, they underwent kinesthetic MI training (from a first-person perspective) for each of the 12 manual positions (Supplementary Appendices 2, 3) included in the present study on 3 consecutive training days (see “Procedures” section). In the kinesthetic MI tasks, the participants were asked to imagining feeling the movements, positions, sensations, etc., without actually performing the hand motor gestures.

The researcher announced a sequence of numbers (2–5 for the left hand, 6–9 for the right hand), which the participants were asked to imagining feeling the movements in the first person using each of the 12 manual motor gestures. To prevent the participants from performing arithmetic tasks to memorize the numbers instead of imagining feeling the movements, an ordinal nomenclature was employed (e.g., second, fourth, sixth, etc.), and the participants were guided during each series of mental tasks so that they performed each opposition task during the 30 s of each series.

#### Action observation

The AO group performed the same motor sequences described for the MI group but by watching videos of each motor gesture, which had the same duration and frequency of movement as for the MI group in the first-person perspective. The sequence of gestures is detailed in the Procedures section. It is therefore that both MI and AO groups performed the same procedure but the first one imagined feeling the movements and the second one observed the movements.

#### Placebo observation group

In the placebo observation (PO) group, participants watched videos representing scenes from a documentary without human agents, a “sham” intervention similar to that conducted by Bassolino et al.^25^.

### Procedures

After giving their consent to partake in the study and prior to the intervention, all participants were given a set of questionnaires. These included a sociodemographic assessment and an evaluation of their physical activity, hand laterality recognition, mental chronometry (MC) and ability to imagine movements. The assessments were designed to have all participants start with the same mental state. The questionnaires were the Spanish-validated version of the International Questionnaire of Physical Activity (IPAQ) and the Spanish-validated version of the Revised Movement Imagery Questionnaire (MIQ-R).

After the baseline assessment was completed, each participant underwent a total of 4 consecutive days of training and assessment. On the first day, the training for the 4 left-hand positions (unimanual positions) was performed (Supplementary Appendix 2). A total of 30 s was spent performing each of the positions (2 min in total), which were performed twice, for a total intervention duration of 4 min. On the second day, the four positions of the right hand were taught in the same manner as the left hand, also with a total intervention duration of 4 min (Supplementary Appendix 2). On the third day, the four positions that included both hands simultaneously (bimanual positions) were taught, spending the same amount of time as the previous days (4 min in total) (Supplementary Appendix 3). On the fourth day, only one evaluation of the 12 sequences of manual motor positions was performed (postintervention evaluation). The duration of the evaluation was approximately 20–25 min. Subsequently, an evaluation was conducted 1 week after the intervention (1-week post), 1 month after the intervention (1-month post) and finally 4 months after the intervention (4-month post). The duration of each follow-up assessment was similar, with 20–25 min spent. During the assessment, the participant never knew the score obtained. No feedback was ever given.

### Outcome measures

#### Primary outcome

##### Accuracy

The accuracy was calculated as follows: each motor gesture in the thumb-opposition tasks involved four fingers in the unimanual gestures and eight fingers in the bimanual gestures (excluding the thumb/s). In the assessment, each subject was asked to place each gesture (one by one) in a real way. In each placement, the time needed by each subject to place the hands gesture was recorded and the hits/success were counted. "Accuracy" refers to the percentage of hits on each of the manual gestures when these were assessed. In unimanual positions, as there are four fingers, each finger that was correct in the position adds up to 25%. Thus, if all four fingers are correctly placed, the "accuracy" is 100%. If there are three fingers placed correctly and one wrong, then the "accuracy" is 75%. If two fingers are positioned correctly and two are positioned incorrectly, then the "accuracy" is 50%, etc. Each success accounted for 12.5% (instead of 25%) of the total score in the bimanual gestures. A participant was considered to have made an error when the two opposing fingers did not perform the gesture or performed a gesture when not appropriate. For example, if a participant performed three correct and five incorrect gestures during a bimanual task (either by not placing the fingers when required or placing the fingers when not required), they would score 3/8 (accuracy = 37.5%) for the task.

#### Secondary outcomes

##### Required time

The time required to position each manual motor gesture from the evaluator’s indication to the participant’s action was recorded in seconds using a stopwatch.

#### Perfect positions

The aim of this variable was to assess the number of positions performed perfectly (i.e., no errors, with a maximum score of 4/4 [100%] for the unimanual and bimanual gestures). Both hands had to perform the position without any error for it to count as perfect. The percentage of perfect positions could range from 0–100%. For example, if a participant obtained a score of 75% in task 1, 100% in task 2, 50% in task 3 and 100% task 4, then only 2 of the 4 positions were perfect, resulting in a score of 2/4 (50%).

#### Baseline outcomes

##### Visual and kinesthetic motor imagery ability

To assess motor imagery ability, we employed the MIQ-R which consists of four movements repeated in two domains (visual and kinesthetic). Depending on the perceived difficulty, participants score the movements from 1 to 7, with one representing the maximum difficulty in creating mental motor imagery and seven representing the least difficulty. The psychometric properties of MIQ-R have been consistently adequate, with Cronbach’s α coefficients ranging above 0.84 for the entire scale, 0.80 for the visual domain and 0.84 for the kinesthetic domain^26^.

#### Mental chronometry

MC is a reliable and widely used tool for recording objective measurements of the ability to create mental motor images^27,28^. For the MC assessment, we used a stopwatch to record the time spent by each participant on imagining the mental tasks included in the MIQ-R. The evaluator issued a command to start imagining the task, and the participant performed a verbal sign once the task had been completed. The time between the two interval commands was recorded, as was the time dedicated by each participant to the real-time execution of the task. The MC values are expressed as the time congruence between the two tasks. The inter-rater intraclass correlation coefficient (ICC) for MC ranged from 0.63–0.95, whereas the ICC for intrasession reliability ranged from 0.95–0.97^27^.

#### Physical activity level

We employed IPAQ to assess the participants’ physical activity level and assign to one of three activity groups (high, moderate and low/sedentary)^29^. The questionnaire’s psychometric properties have been accepted for use in studies that measure physical activity; IPAQ has a reliability of approximately 0.65 (r = 0.76; 95% CI 0.73–0.77)^30^.

#### Laterality recognition task

For the hand laterality recognition task, we evaluated two aspects: (1) the percentage of correct answers for laterality discrimination, which is the ability to recognize whether a body part belongs to the right or left side of the body^31^ and (2) the response time employed by the participants for the discrimination task or cognitive judgment. We employed the Recognise Online application designed and developed by the NOI group (Neuro Orthopaedic Institute)^32^ whose reliability has been previously established in populations with and without chronic pain^32^. The ICC response time was described for only the feet (ICC 0.63–0.75) and trunk (ICC 0.51–0.91).

### Data analysis

We employed the Statistical Package for the Social Sciences (SPSS 23.00, IBM, Chicago, IL, USA) for the data analysis, employing a confidence interval of 95% and considering all variables with a *p* value < 0.05 as statistically significant. We used descriptive statistics to summarize the data for continuous variables, which are presented as mean ± standard deviation with 95% confidence intervals. The categorical variables are presented as absolute numbers or relative frequencies (percentages). To compare the categorical variables, we employed a chi-squared test with residual analysis. The normal distribution of all primary and secondary measures was assessed using the Shapiro–Wilk test. We performed a repeated measures analysis of variance (ANOVA) to study the effect of the interparticipant factor “intervention group” (consisting of three categories: AO, MI and PO) and the intraparticipant factor “time” (consisting of four categories: postintervention, 1 week postintervention, 1 month postintervention and 4 months postintervention) on the dependent variables. We calculated the partial eta squared (ƞ_p_^2^) as a measure of the effect size (strength of association) for each main effect and interaction in the ANOVAs, with 0.01–0.059 representing a small effect, 0.06–0.139 a medium effect and > 0.14 a large effect. We performed a post hoc analysis with Bonferroni correction in the case of significant ANOVA findings for multiple comparisons between variables. We calculated the effect size (Cohen’s *d*) for the main variables, considering 0.20–0.49, 0.50–0.79 and > 0.80 to be small, medium and large effect sizes, respectively^33^. In addition, a secondary analysis was conducted to determine if the ability to imagine movements could have an impact on the results obtained, especially for the MI group. For this purpose, we calculated the median score for MI group of the participants in the MIQ-R questionnaire and classified the participants into “good imagers” (those above median) or “poor imagers” (those below median). The same analysis that was done for the MI group was also done with the other two groups (AO and PO).

### Ethical approval

All procedures were approved by the Human Research Ethics Committee of the La Salle University Center for Advanced Studies (CSEULS-PI-013/2019). The study was registered in the United States Randomized Trials Register on clinicaltrial.gov (trial registry number: NCT03769974).

### Informed consent and ethics

All participants granted their informed written consent prior to inclusion and were provided an explanation of the study procedures, which were planned under the ethical standards of the Helsinki Declaration.

## Results

A total of 45 asymptomatic participants were included and randomly allocated to 3 groups of 15 participants each. No adverse events or loss to follow-up were reported for any group. There were no statistically significant differences in demographic data prior to the intervention between the groups and the self-reported variables, except for body mass index (*p* = 0.02) (Table 1).

**Table 1:** Descriptive statistics for the sociodemographic and self-reported data.

Measures	MI (n = 15)	AO (n = 15)	PO (n = 15)	*p-*value
Age (year)	32.0 ± 12.5	32.9 ± 14.0	29.3 ± 6.7	0.66
BMI (kg/m^2^)	23.4 ± 2.2	20.8 ± 1.9	23.7 ± 2.4	0.02*
MIQ-R	48.3 ± 6.6	50.4 ± 5.1	48.6 ± 6.5	0.59
MIQ-RK	23.6 ± 3.6	24.2 ± 4.6	24.4 ± 3.1	0.84
MIQ-RV	24.6 ± 3.4	26.2 ± 1.7	24.2 ± 3.6	0.16
MC	1.28 ± 0.3	1.13 ± 0.2	1.42 ± 0.4	0.13
K-MC	1.36 ± 0.4	1.22 ± 0.3	1.45 ± 0.5	0.16
V-MC	1.19 ± 0.3	1.04 ± 0.3	1.41 ± 0.5	0.06
LRT %Total	80.3 ± 10.7	85.0 ± 9.1	81.6 ± 6.9	0.35
LTT %Right Hand	78.0 ± 16.5	84.6 ± 9.9	84.0 ± 9.1	0.27
LRT %Left Hand	82.7 ± 7.9	84.0 ± 9.1	79.3 ± 11.6	0.40
LRT Time	2.4 ± 0.8	2.2 ± 0.3	2.1 ± 0.3	0.35
LRT Right-Hand Time	2.2 ± 0.8	2.2 ± 0.4	2.2 ± 0.5	0.96
LRT Left-Hand Time	2.5 ± 1.1	2.2 ± 0.3	2.0 ± 0.7	0.18
IPAQ	2,879.5 ± 1,443.1	2,589.2 ± 1,238.6	2077.9 ± 1,164.8	.430
IPAQ-Level				0.14
Low	0 (0)	0 (0)	0 (0)	
Moderate	10 (66.7)	10 (66.7)	14 (93.3)	
High	5 (33.3)	5 (33.3)	1 (6.7)	
Sex				0.91
Male	9 (60)	8 (53.3)	9 (60)	
Female	6 (40)	7 (46.7)	6 (40)	
Educational level				0.88
Secondary education	3 (20)	4 (26.7)	4 (26.7)	
College education	12 (80)	11 (73.3)	11 (73.3)	
Dominant hand				0.34
Right	14 (93.3)	15 (100)	13 (86.7)	
Left	1 (6.7)	0 (0)	2 (13.3)	

Values are presented as mean ± standard deviation or number (%).

*AO* action observation, *BMI* body mass index, *IPAQ* International Physical Activity Questionnaires, *K* kinesthetic subscale, *LRT* laterality recognition task, *MI* motor imagery, *MIQ-R* movement imagery questionnaire-revised, *MC* mental chronometry, *PO* placebo observation group, *V* visual subscale, %, successful.

### Primary outcome

#### Accuracy

In terms of the left hand, the ANOVA revealed significant changes during group × time (*F* = 2.84, *p* = 0.023, ƞ_p_^2^ = 0.119) and time (*F* = 21.19, *p* < 0.001, ƞ_p_^2^ = 0.335). The post hoc analysis showed that the MI and AO groups showed statistically significant differences compared with the PO postintervention (*p* < 0.001; *d* = 2.65, and *d* = 4.78, respectively), 1 week postintervention (*p* < 0.001; *d* = 1.94, and *d* = 4.45, respectively) and 1 month postintervention (*p* < 0.001; *d* = 1.58, and *d* = 2.90, respectively), with a large effect size. However, only the AO group showed significant differences compared with the PO group at 4 months postintervention, with a large effect size (*p* < 0.001, *d* = 2.25). The AO was also superior to the MI group at 1 week (*p* = 0.034, *d* = 0.99), 1 month (*p* = 0.016, *d* = 0.98) and 4 months (*p* = 0.003, *d* = 1.10) postintervention, with a large effect size. However, there were no differences between the 2 mental practice groups postintervention (*p* > 0.05). The intragroup differences are summarized in Table 2.

**Table 2:** Intragroup differences in the accuracy (%) outcome measure.

Measure	Group	Mean ± SD				Mean difference (95% CI); effect size (*d*) (a) post—1 week (b) post—1 month (c) post—4 months
		Post	1 week	1 month	4 months	
Left hand	PO	56.6 ± 12.6	51.6 ± 14.2	52.9 ± 12.4	45.8 ± 9.9	(a) 5.0 (− 4.6 to 14.6); *d* = 0.37 (b) 3.7 (− 6.9 to 14.4); *d* = 0.29 (c) 10.8 (− 4.3 to 26.0); *d* = 0.95
	MI	93.7 ± 15.3	84.1 ± 18.8	77.9 ± 18.4	59.6 ± 22.5	(a) 9.6 (− 0.1 to 19.2); *d* = 0.56 (b) 15.8* (5.1 to 26.5); *d* = 0.93 (c) 34.1** (18.9 to 49.3); *d* = 1.77
	AO	99.5 ± 1.6	97.5 ± 3.1	95.0 ± 16.2	84.1 ± 21.8	(a) 2.1 (− 7.5 to 11.7); *d* = 0.81 (b) 4.5 (− 6.1 to 15.2); *d* = 0.39 (c) 15.4* (0.2 to 30.6); *d* = 0.99
Right hand	PO	53.7 ± 17.8	55.4 ± 15.2	53.7 ± 12.4	40.8 ± 11.7	(a) − 1.6 (− 8.3 to 4.9); *d* = − 0.1 (b) 0 (− 10.0 to 10.0); *d* = 0 (c) 12.9* (0.9 to 24.9); *d* = 0.85
	MI	78.7 ± 22.5	77.9 ± 22.0	74.1 ± 21.7	53.3 ± 23.8	(a) 0.8 (− 5.7 to 7.4); *d* = 0.03 (b) 4.5 (− 5.5 to 14.6); *d* = 0.2 (c) 25.4** (13.4 to 37.4); *d* = 1.09
	AO	95.0 ± 8.2	92.9 ± 8.1	92.0 ± 15.9	85.41 ± 18.7	(a) 2.08 (− 4.5 to 8.7); *d* = 0.25 (b) 2.97 (− 7.2 to 13.0); *d* = 0.23 (c) 9.6 (− 2.4 to 21.5); *d* = 0.66
Both hands	PO	57.9 ± 11.6	49.1 ± 21.8	43.7 ± 6.8	41.7 ± 5.0	(a) 8.7 (− 2.2 to 19.7); *d* = 0.5 (b) 14.1** (6.3 to 22.0); *d* = 1.49 (c) 16.2** (6.8 to 25.5); *d* = 1.81
	MI	72.0 ± 20.4	82.5 ± 19.9	66.2 ± 19.8	61.4 ± 19.5	(a) − 10.4 (− 21.4 to 0.6); *d* = − 0.52 (b) 5.8 (− 1.9 to 13.7); *d* = 0.28 (c) 10.6* (1.3 to 19.9); *d* = 0.53
	AO	94.7 ± 5.6	90.8 ± 13.7	89.7 ± 9.8	78.0 ± 15.8	(a) 3.9 (− 7.0 to 14.9); *d* = 0.37 (b) 5.0 (− 2.7 to 12.9); *d* = 0.62 (c) 16.8** (7.4 to 26.1); *d* = 1.40

*p < 0.05; **p < 0.001.

*AO* action observation group, *CI* confidence interval, *m* month, *MI* motor imagery group, *PO* placebo observation group, *SD* standard deviation, *w* week.

In terms of the right hand, the ANOVA revealed significant changes during group × time (*F* = 2.39, *p* = 0.048, ƞ_p_^2^ = 0.102) and time (*F* = 24.12, *p* < 0.001, ƞ_p_^2^ = 0.365). The post hoc analysis showed that the MI and AO groups showed statistically significant differences compared with the PO group postintervention (*p* < 0.01; *d* = 1.23, and *d* = 2.97, respectively), 1 week postintervention (*p* < 0.01; *d* = 1.18, and *d* = 3.06, respectively) and 1 month postintervention (*p* < 0.01; *d* = 1.08, and *d* = 2.44, respectively), with a large effect size. However, only the AO group showed significant differences compared with the PO group at 4 months postintervention, with a large effect size (*p* < 0.001, *d* = 2.85). The AO group was also superior to the MI group postintervention (*p* = 0.04, *d* = 0.95), 1 week postintervention (*p* = 0.045, *d* = 0.90), 1 month postintervention (*p* = 0.02, *d* = 0.94) and 4 months postintervention (*p* < 0.001, *d* = 1.49), with a large effect size. The intragroup differences are summarized in Table 2.

**Figure 1:**
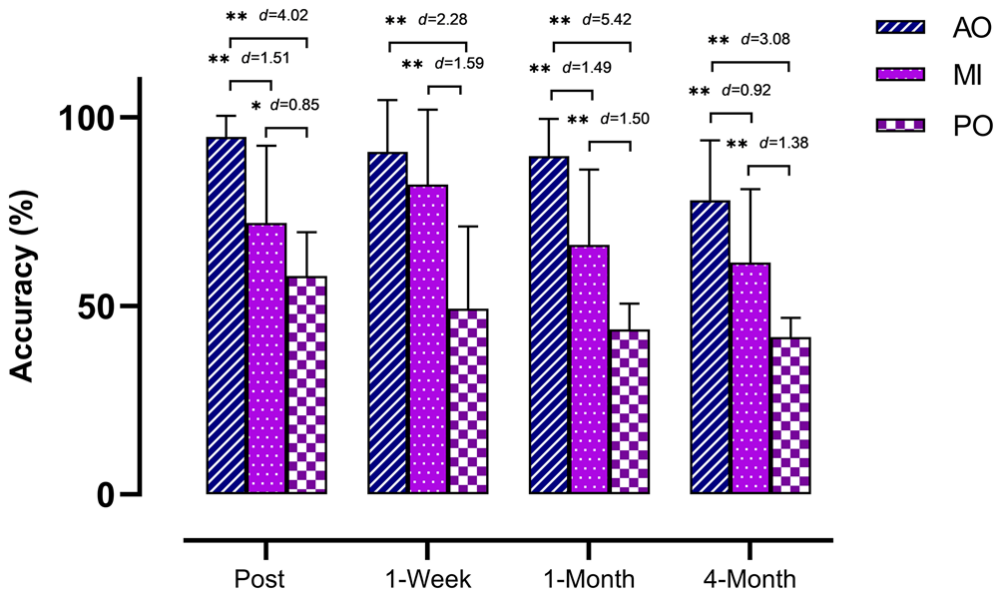
Between-group differences in accuracy (%) outcome measure regarding bimanual gestures. *p < 0.05; ** p < 0.001; *AO* action observation, *MI* motor imagery, *PO* placebo observation group, *d* d of Cohen.

In terms of both hands, the ANOVA revealed significant changes during group × time (*F* = 3.03, *p* = 0.017, ƞ_p_^2^ = 0.126) and time (*F* = 19.19, *p* < 0.001, ƞ_p_^2^ = 0.314). The post hoc analysis showed that the MI and AO groups showed statistically significant differences compared with the PO post postintervention (*p* < 0.05; *d* = 0.85 and *d* = 4.02, respectively), 1 week postintervention (*p* < 0.001; *d* = 1.59 and *d* = 2.28, respectively), 1 month postintervention (*p* < 0.001; *d* = 1.50 and *d* = 5.42, respectively) and 4 months postintervention (*p* < 0.001; *d* = 1.38 and *d* = 3.08, respectively), with a large effect size. The AO group was also superior to the MI group postintervention (*p* < 0.001, *d* = 1.51), 1 month postintervention (*p* < 0.001, *d* = 1.49) and 4 months postintervention (*p* = 0.012, *d* = 0.92), with a large effect size. There were no significant differences 1 week postintervention between the MI and AO groups (*p* > 0.05) (Fig. 1). The intragroup differences are summarized in Table 2.

### Secondary outcomes

#### Required time

In terms of the left hand, the ANOVA revealed significant changes during group × time (*F* = 4.75, *p* = 0.001, ƞ_p_^2^ = 0.185) and time (*F* = 3.56, *p* = 0.029, ƞ_p_^2^ = 0.078). The post hoc analysis showed that the MI group spent significantly more time than the AO group at 1 month (*p* < 0.001, *d* = 1.46) and 4 months (*p* = 0.003; *d* = 1.07) postintervention, with a large effect size. The AO group needed more time than the PO group only at 4 months postintervention, with a large effect size (*p* = 0.025, *d* = 1.47). The MI group spent significantly more time than the PO group at 1 week (*p* = 0.021, *d* = 1.04), 1 month (*p* = 0.009, *d* = 1.03) and 4 months (*p* < 0.001, *d* = 2.21) postintervention, with a large effect size. The intragroup differences are shown in Table 3.

**Table 3:** Intragroup differences in requested time outcome measurement.

Measure	Group	Mean ± SD				Mean difference (95% CI); effect size (*d*) (a) post—1 week (b) post—1 month (c) post—4 months
		Post	1 week	1 month	4 months	
Left hand, s	PO	1.72 ± 0.9	1.19 ± 0.4	1.42 ± 0.3	1.07 ± 0.1	(a) 0.53* (0.0 to 1.0); *d* = 0.76 (b) 0.3 (− 0.2 to 0.8); *d* = 0.44 (c) 0.65* (0.1 to 1.2); *d* = 1.01
	MI	1.94 ± 0.7	1.72 ± 0.6	1.92 ± 0.5	2.32 ± 0.7	(a) 0.21 (− 0.3 to 0.7); *d* = 0.33 (b) 0.01 (− 0.5 to 0.5); *d* = 0.03 (c) − 0.38 (− 0.9 to 0.1); *d* = − 0.54
	AO	1.39 ± 0.5	1.35 ± 0.5	1.24 ± 0.3	1.62 ± 0.5	(a) 0.03 (− 0.5 to 0.5); *d* = 0.08 (b) 0.15 (− 0.3 to 0.6); *d* = 0.36 (c) − 0.23 (− 0.8 to 0.3); *d* = − 0.46
Both hands, s	PO	2.82 ± 1.2	2.84 ± 1.3	2.18 ± 0.8	1.38 ± 0.6	(a) − 0.02 (− 1.0 to 0.9); *d* = − 0.02 (b) 0.64 (− 0.2 to 1.5); *d* = 0.95 (c) 1.43* (0.4 to 2.4); *d* = 2.6
	MI	4.54 ± 1.8	3.48 ± 1.5	3.49 ± 1.4	3.5 ± 1.1	(a) 1.06* (0.0 to 2.0); *d* = 0.64 (b) 1.05* (0.1 to 1.9); *d* = 0.65 (c) 1.04* (0.0 to 2.0); *d* = 0.69
	AO	1.91 ± 0.7	1.75 ± 0.6	1.81 ± 0.5	2.4 ± 0.8	(a) 0.16 (− 0.8 to 1.1); *d* = 0.24 (b) 0.09 (− 0.7 to 0.9); *d* = 0.16 9c) − 0.48 (− 1.5 to 0.5); *d* = − 0.65

*p < 0.05; **p < 0.001.

*AO* action observation group, *CI* confidence interval, *m* month, *MI* motor imagery group, *PO* placebo observation group, *s* seconds, *SD* standard deviation, *w* week.

In terms of the right hand, the ANOVA revealed no significant differences in time (*F* = 1.68, *p* = 0.18, ƞ_p_^2^ = 0.038) or in group × time (*F* = 2.10, *p* = 0.071, ƞ_p_^2^ = 0.091).

In terms of both hands, the ANOVA revealed significant changes during group × time (*F* = 5.02, *p* < 0.001, ƞ_p_^2^ = 0.193) and time (*F* = 4.72, *p* = 0.004, ƞ_p_^2^ = 0.101). The post hoc analysis showed that the MI group spent significantly more time than the AO group postintervention (*p* < 0.001, *d* = 1.90), 1 week (*p* = 0.002, *d* = 1.44), 1 month postintervention (*p* < 0.001, *d* = 1.58) and 4 months postintervention (*p* = 0.004, *d* = 1.11), with a large effect size. The AO group needed more time than the PO group only at 4 months postintervention, with a large effect size (*p* = 0.009, *d* = 1.34). The MI group spent significantly more time than the PO group postintervention (*p* = 0.003, *d* = 1.11), 1 month postintervention (*p* = 0.002, *d* = 1.13) and 4 months postintervention (*p* < 0.001, *d* = 2.38), with a large effect size. The intragroup differences are shown in Table 3.

#### Perfect positions

In terms of the left hand, the ANOVA revealed significant changes during group × time (*F* = 4.89, *p* = 0.001, ƞ_p_^2^ = 0.189) and time (*F* = 19.13, *p* < 0.001, ƞ_p_^2^ = 0.313). The post hoc analysis showed that both the MI and AO groups showed statistically significant differences compared with the PO group postintervention (*p* < 0.001; *d* = 3.50 and *d* = 5.61, respectively), 1 week postintervention (*p* < 0.001; *d* = 2.09 and *d* = 5.59, respectively) and 1 month postintervention (*p* < 0.01; *d* = 1.44 and *d* = 3.60, respectively), with a large effect size. However, only the AO group showed significant differences compared with the PO group at 4 months postintervention, with a large effect size (*p* < 0.001, *d* = 2.32). The AO group was also superior to the MI group at 1 week (*p* = 0.027, *d* = 0.88), 1 month (*p* = 0.001, *d* = 1.29) and 4 months (*p* = 0.003, *d* = 1.09) postintervention, with a large effect size. However, there were no differences between the two mental practice groups postintervention (*p* > 0.05). The intragroup differences are summarized in Table 4.

**Table 4:** Intragroup differences in perfect positions (%) outcome measure.

Measure	Group	Mean ± SD				Mean difference (95% CI); effect size (*d*) (a) post—1 week (b) post—1 month (c) post—4 months
		Post	1 week	1 month	4 months	
Left hand	PO	11.6 ± 20.8	10.0 ± 15.8	8.3 ± 15.4	5.0 ± 10.3	(a) 1.6 (− 12.9 to 16.2); *d* = 0.08 (b) 3.3 (− 17.7 to 24.4); *d* = 0.18 (c) 6.6 (− 16.8 to 30.1); *d* = 0.40
	MI	88.3 ± 22.8	66.6 ± 34.9	48.3 ± 35.9	30.0 ± 33.0	(a) 21.6* (7.0 to 36.2); *d* = 0.73 (b) 40.0** (18.8 to 61.1); *d* = 1.33 (c) 58.3** (34.8 to 81.8); *d* = 2.05
	AO	98.3 ± 6.4	90.0 ± 12.6	90.0 ± 28.0	68.3 ± 37.1	(a) 8.3 (− 6.2 to 22.9); *d* = 0.83 (b) 8.3 (− 12.7 to 29.4); *d* = 0.40 (c) 30.0* (6.5 to 53.5); *d* = 1.12
Right hand	PO	18.3 ± 29.0	18.3 ± 27.5	11.6 ± 15.9	0.0 ± 0.0	(a) 0.0 (− 12.7 to 12.7); *d* = 0 (b) 6.6 (− 11.2 to 24.6); *d* = 0.28 (c) 18.3 (− 2.9 to 36.6); *d* = 0.89
	MI	58.3 ± 44.9	60.0 ± 36.3	51.6 ± 39.5	26.6 ± 34.6	(a) − 1.6 (− 14.4 to 11.0); *d* = − 0.04 (b) 6.6 (− 5.5 to 14.6); *d* = 0.15 (c) 31.6*(10.4 to 52.9); *d* = 0.79
	AO	88.3 ± 18.6	83.3 ± 18.1	83.3 ± 24.4	73.3 ± 27.5	(a) 5.0 (− 7.7 to 17.7); *d* = 0.27 (b) 5.0 (− 12.9 to 22.9); *d* = 0.23 (c) 15.0 (− 6.2 to 36.2); *d* = 0.63
Both hands	PO	10.0 ± 15.8	0.0 ± 0.0	0.0 ± 0.0	0.0 ± 0.0	(a) 10.0 (− 3.9 to 23.9); *d* = 0.89 (b) 10.0 (− 6.6 to 26.6); *d* = 0.89 (c) 10.0 (− 6.4 to 26.4); *d* = 0.89
	MI	46.6 ± 36.4	40.0 ± 32.4	30.0 ± 34.3	23.3 ± 34.6	(a) 6.6 (− 7.2 to 20.5); *d* = 0.19 (b) 16.6* (0.5 to 33.2); *d* = 0.46 (c) 23.3* (6.9 to 39.7); *d* = 0.65
	AO	85.0 ± 15.8	78.3 ± 22.8	68.3 ± 24.0	41.6 ± 27.8	(a) 6.6 (− 7.2 to 20.5); *d* = 0.34 (b) 16.7* (0.5 to 33.3); *d* = 0.82 (c) 43.3** (26.9 to 59.7); *d* = 1.91

*p < 0.05; **p < 0.001.

*AO* action observation group, *CI* confidence interval, *m* month, *MI* motor imagery group, *PO* placebo observation group, *SD* standard deviation, *w* week.

In terms of the right hand, the ANOVA revealed significant changes during time (*F* = 15.05, *p* < 0.001, ƞ_p_^2^ = 0.264) but not during group × time (*F* = 1.33, *p* = 0.248, ƞ_p_^2^ = 0.06). The post hoc analysis showed that the MI and AO groups showed statistically significant differences compared with the PO group at all assessment times, with a large effect size (*p* < 0.01, *d* > 0.8). The AO group was also superior to the MI group postintervention (*p* = 0.048, *d* = 0.87), 1 month postintervention (*p* = 0.012, *d* = 0.81) and 4 months postintervention (*p* < 0.001, *d* = 1.49), with a large effect size. However, there were no differences between the MI and AO groups at 1 week postintervention (*p* > 0.05). The intragroup differences are summarized in Table 4.

**Figure 2:**
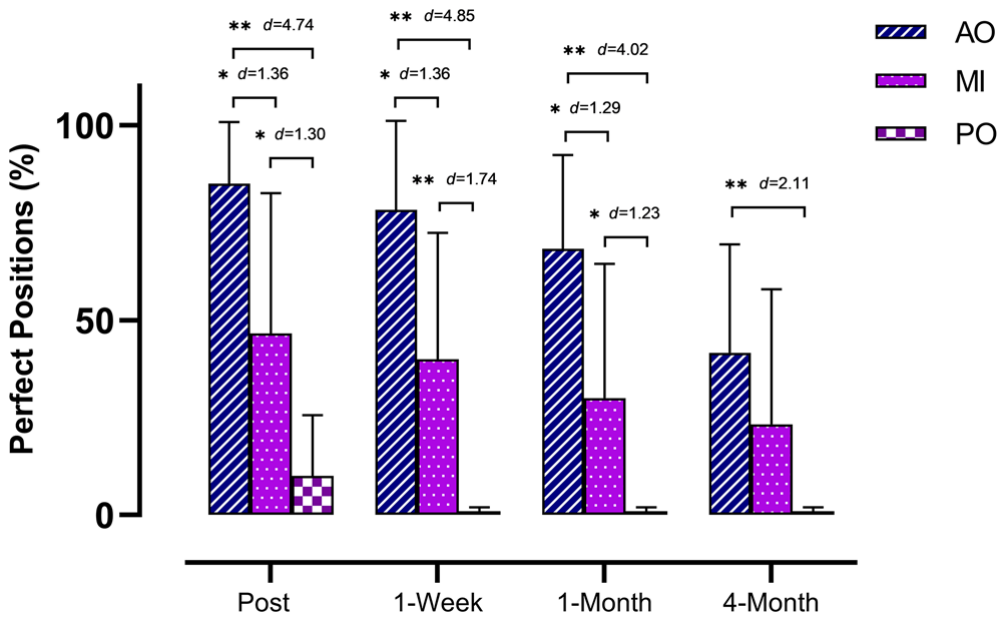
Between-group differences in perfect positions (%) outcome measure regarding bimanual gestures. *p < 0.05; **p < 0.001; *AO* action observation, *MI* motor imagery, *PO* placebo observation group, *d* d of Cohen.

In terms of both hands, the ANOVA revealed significant changes during group × time (*F* = 5.60, *p* < 0.001, ƞ_p_^2^ = 0.211) and time (*F* = 27.37, *p* < 0.001, ƞ_p_^2^ = 0.395). The post hoc analysis showed that the MI and AO groups showed statistically significant differences compared with the PO group postintervention (*p* < 0.01; *d* = 1.30 and *d* = 4.74, respectively), 1 week postintervention (*p* < 0.001; *d* = 1.74 and *d* = 4.85, respectively) and 1 month postintervention (*p* < 0.01; *d* = 1.23 and *d* = 4.02, respectively), with a large effect size. However, only the AO group showed significant differences compared with the PO group 4 months postintervention, with a large effect size (*p* < 0.001, *d* = 2.11). The AO group was also superior to the MI group postintervention (*p* < 0.001, *d* = 1.36), 1 week postintervention (*p* < 0.001, *d* = 1.36) and 1 month postintervention (*p* = 0.012, *d* = 1.29), with a large effect size. However, there were no significant differences 4 months postintervention between the MI and AO groups (*p* > 0.05) (Fig. 2). The intragroup differences are summarized in Table 4.

#### Analysis according to the ability to imagine movements

In the MI group, based on the median score achieved in the MIQ-R questionnaire (Md = 50 points), the participants were classified into “good imagers” (those above median; n = 8) or “poor imagers” (those below median; n = 7).

#### Accuracy

Regarding the left hand, the ANOVA revealed significant changes during time (*F* = 7.71, *p* = 0.005, ƞ_p_^2^ = 0.68) but not in group × time interaction (*F* = 2.04, *p* = 0.166, ƞ_p_^2^ = 0.358).

For the right hand, the ANOVA revealed significant changes during time (*F* = 9.82, *p* = 0.002, ƞ_p_^2^ = 0.728) but not in group × time interaction (*F* = 2.83, *p* = 0.087, ƞ_p_^2^ = 0.436).

Finally, regarding both hands, the ANOVA revealed significant changes during time (*F* = 4.41, *p* = 0.029, ƞ_p_^2^ = 0.546) but not in group × time interaction (*F* = 0.217, *p* = 0.883, ƞ_p_^2^ = 0.056).

#### Time required

Regarding the left hand, the ANOVA revealed significant changes during time (*F* = 5.78, *p* = 0.013, ƞ_p_^2^ = 0.61) but not in group × time interaction (*F* = 0.876, *p* = 0.483, ƞ_p_^2^ = 0.193).

In relation to the right hand, the ANOVA did not reveal significant changes during time (*F* = 1.60, *p* = 0.245, ƞ_p_^2^ = 0.303) and not in group × time interaction (*F* = 0.43, *p* = 0.74, ƞ_p_^2^ = 0.105).

For both hands, the ANOVA revealed significant changes during time (*F* = 4.23, *p* = 0.032, ƞ_p_^2^ = 0.535) but not in group × time interaction (*F* = 3.11, *p* = 0.071, ƞ_p_^2^ = 0.459).

#### Perfect positioning

Regarding left hand, the ANOVA revealed significant changes during time (*F* = 9.61, *p* = 0.002, ƞ_p_^2^ = 0.724) but not in group × time interaction (*F* = 2.86, *p* = 0.085, ƞ_p_^2^ = 0.439).

For the right hand, the ANOVA revealed significant changes during time (*F* = 6.6, *p* = 0.001, ƞ_p_^2^ = 0.43) but not in group × time interaction (*F* = 1.82, *p* = 0.18, ƞ_p_^2^ = 0.738).

Finally, regarding both hands, the ANOVA revealed significant changes during time (*F* = 4.53, *p* = 0.02, ƞ_p_^2^ = 0.545) but not in group × time interaction (*F* = 2.21, *p* = 0.011, ƞ_p_^2^ = 0.559).

#### Action observation and placebo group

The ANOVA revealed no difference in time or group × time interaction for either hand or any of the variables. The median score for each group was Md = 52 for the AO group, and Md = 51 for de PO group.

## Discussion

The primary objective of the present study was to assess the short to medium-term impact of MI and AO in isolation on the motor learning of a sequence of thumb-opposition tasks of increasing complexity in terms of accuracy compared with a placebo intervention. The secondary objectives were to evaluate the required time and the percentage of perfect positions. In addition, we also aimed to assess the effects on motor learning based on the ability to imagine movements in order to verify whether good imagers showed greater benefits than poor imagers accordingly to each intervention.

The results of the present study showed that the AO group had significantly higher accuracy than the MI or PO group until at least 4 months after the mental practice intervention in isolation, an aspect that had not been shown in scientific literature until these results. In the study by Gatti et al.^15^ only one intervention session was performed; thus, only the rapid phase of the motor learning process was applied. The study participants had to learn a complex and unusual motor task that involved moving the right hand and foot in the same angular direction, while simultaneously moving the left hand and foot in an opposite angular direction. The authors employed a kinematics analysis to assess the motor learning process, the results of which are in line with those of our study, i.e., the authors found that AO was more effective than MI. González-Rosa et al.^16^ also found that AO was more effective than MI in promoting the early learning of a new complex coordination task. In patients with chronic neck pain, Cuenca-Martínez et al.^34^ showed that AO intervention in isolation showed the strongest results improving cervical joint position sense in comparison with MI and placebo group.

The results of the present study support these findings and show that AO is more effective than MI in motor learning until at least 4 months after the motor training session for unimanual motor positions and until at least 1 month later in the bimanual positions. It would have been interesting to conduct neuroimaging studies to evaluate the neurophysiological functional connections caused by brain training and motor learning.

These studies focused on the study of motor learning through the movement representation techniques in isolation. However, several studies have evaluated the effect of mental practice in combination with physical practice to also evaluate the process of motor learning. For example, Cuenca-Martínez et al.^35^ found that AO plus physical practice caused faster changes in lumbopelvic motor control compared with only physical practice but not, in comparison with MI plus physical practice in asymptomatic participants. In fact, it seems that combining mental practice and physical practice is likely to minimize the differences between the two movement representation techniques (MI and AO). We did not enter differences between AO and MI groups either by adding physical practice with respect to improving strength^36^.

In terms of the time required, the results showed that the MI group required significantly more time than the PO and AO groups to remember and perform the left-hand and two-handed gestures. At this point, the question becomes, why does AO show different results than MI? Gatti et al.^15^ have argued that AO has a greater impact than MI due to several factors. First, the mirror neuron system functions more accurately and adequately through observation. For example, the ventral premotor cortex, an area widely involved in the planning of voluntary movement, receives afferences from the visual cortex. AO might therefore cause greater neurophysiological functional activation than imagination does.

The relationship between learning and required time has been extensively explored in the literature. Some models in this regard have found an increase in the speed of task execution as a skill learning is consolidated, which could explain the results in favor of the AO group^37^. However, a surprising result is that the PO group took less required time to perform the gesture, although they made many more mistakes. In that sense, some studies have shown that people with a lower skill may have a higher frustration when executing the task, as well as a lower motivation, which leads them not to take the task seriously, and may explain the lower time spent on it^38^.

In addition, the act of imagining can vary among individuals and could therefore be related to associated variables such as the physical activity level, the ability to imagine movements, the complexity of the task to be imagined, the time spent imagining, the effort required for the task and the vividness and controllability of the image^8,36–39^. Therefore, although the AO group had an exact, precise, unambiguous, and specific reference model for the required motor positions, the MI group had only its own capacity to imagine movements to perform the brain training, so the insufficient mental engagement could be possible in MI group. This fact could also be relevant in explaining the findings of this study.

We also hypothesize that fatigue due to MI is another possible factor explaining the observed differences. Roure et al.^43^ and Guillot et al.^44^ have reported that mental practice causes mental fatigue and difficulty maintaining attention. Future research should compare different MI dosages to assess effects with respect to AO training but always monitoring fatigue because it can be an important physical condition to take into consideration. The loss of attention might therefore be greater in the MI group than in the AO group, which could be an important variable in motor learning. Buccino^12^ argued that MI has certain intrinsic limits that AO does not exhibit because MI is a more demanding tool than AO in terms of attention and concentration. This argument agrees with the hypotheses proposed in the present study in the comparison between the two sensorimotor neuro-training tools.

Regarding the aimed to assess the effects on motor learning based on the ability to imagine movements, one of the most interesting hypotheses is whether participants with greater ability to perform mental motor images can obtain more benefits from the intervention, especially from MI. However, the results of this analysis showed no difference between “good imagers” and “poor imagers” in terms of accuracy, time required or totally correct positions.

Previous studies have shown greater benefits in MI in subjects with greater ability to imagine movements, such as Robin et al.^8^, in relation to motor performance in a specific tennis gesture. However, it is necessary to highlight that all the participants of our study presented high levels to imagine movements, especially in a kinesthetic manner. It is possible that both groups (better and poorer imagers) had a good ability to imagine movements, which could explain the absence between-groups differences.

### Potential applications

We analyzed the study’s results a theoretical viewpoint and a from a functional viewpoint and from. In terms of functionality, the assessment 1 week postintervention has greater importance. In light of the results of the more complex tasks for the perfect positions, AO was better than MI; however, AO was not superior in terms of accuracy. From a theoretical viewpoint and to answer the question, “which mental practice tool in isolation has a greater and more lasting potential in motor learning?”, it appears that AO training is superior to MI with minimal training. However, it would be interesting to perform this theoretical comparison by training the motor imagery group beforehand or by increasing the intervention load.

From a functional point of view, AO could be employed both in isolation and in combination with real practice to learn gestures and motor positions widely demanded in several fields, such as music (position of chords or notes), sport (gestures, grips, skill acquisition), neurorehabilitation, improvement of surgical techniques and in communication processes such as sign language, which uses fixed manual positions for word exchange. In addition, tools such as AO can enable a motor learning (or relearning) process and maintain it over time and can have a clinical effect on patients who, for whatever reason (e.g., surgery, immobilization.), cannot move in real-time and should be employed to improve patient outcomes. MI could also be used but it appears that the effect is significantly less than AO training. However, the main advantage of MI is that scenarios can be changed and adapted to the patient's context.

### Limitations

This study presents several limitations. First, an important objective of the present study was to observe the differences between mental practice groups with a minimal intervention. However, Hinshaw^45^ argued that the optimal time for obtaining the greatest benefits with MI is between 10 and 15 min. In the present study, the duration of the MI intervention was shorter, which might have been insufficient to obtain the full potential of MI. Second, AO and MI techniques, although sharing a large network of neurophysiological activity, these are not the same. During AO training, dependent areas of the visual cortex are activated with greater intensity compared with MI in a kinesthetic manner. However kinesthetic MI includes a greater component of somatosensory stimulation such as the dorsal column-medial lemniscus pathway. It would have been very interesting, and this should be considered a major limitation, to include a fourth group that only performed visual MI to be able to compare with AO because of their convergence in the neurophysiology underlying both techniques. Third, we did not evaluate the perceived fatigue, which could have been an interesting variable for explaining the results of the present study. Fourth, it would have been interesting to evaluate the autonomic variables during training to evaluate mental effort indirectly, especially in the MI group. Fifth, we did not measure the participants’ perceived difficulty in learning each gesture nor the vividness of the intervention. The relationship between these variables and the study’s results would have been interesting to know. Sixth, the AO group showed a greater ability to imagine movements visually than the other groups, which should be taken into account. Seventh, it was not possible to control the intervention to the participants during the follow up, so it is not possible to state categorically that everyone performed the task and learning adequately, which could influence the differences found. Finally, we could have used more functional motor gestures than just thumb to finger-opposition tasks to operationalize motor learning.

## Conclusions

Based on the results obtained, AO training was superior to MI until at least 4 months postintervention in terms of accuracy and perfect positions for the unimanual gestures, and until at least 1 month postintervention in terms of perfect positions for the bimanual gestures. However, in terms of accuracy for the bimanual gestures, AO was not superior to MI at only 1 week postintervention. The AO group also required less time than the MI group to remember and perform the manual positions. Both AO and MI were superior to the placebo intervention until at least 1 month postintervention, and only AO was superior at 4 months. MI was never superior to AO training. Finally, “good imagers” did not obtain any better results than the “poor imagers” on any outcome measure in this study probably because all participants had a high ability to imagine movements. AO could be employed to learn gestures and motor positions, so could be a useful tool for enable a motor learning in the short to medium-term. MI could also be used but it appears that the effect is significantly less than AO training.

## Supplementary information

Supplementary file1.

## Data Availability

Study data is available upon request from the authors.
