# Supplementary material for: Effects of movement representation techniques on motor learning of thumb-opposition tasks
Source: Sci Rep. 2020 Jul 23;10:12267. doi: 10.1038/s41598-020-67905-7 (PMC7378061; doi:10.1038/s41598-020-67905-7)
Supplement: Supplementary file 1 — Supplementary file1. [file 41598_2020_67905_MOESM1_ESM.pdf]

Title: Effects of Movement Representation Techniques on Motor Learning of Thumb-Opposition Tasks

Author list: Ferran Cuenca-Martínez, Luis Suso-Martí, Jose Vicente León-Hernández, Roy La Touche

Appendix 1. Numerical assignment to hand fingers

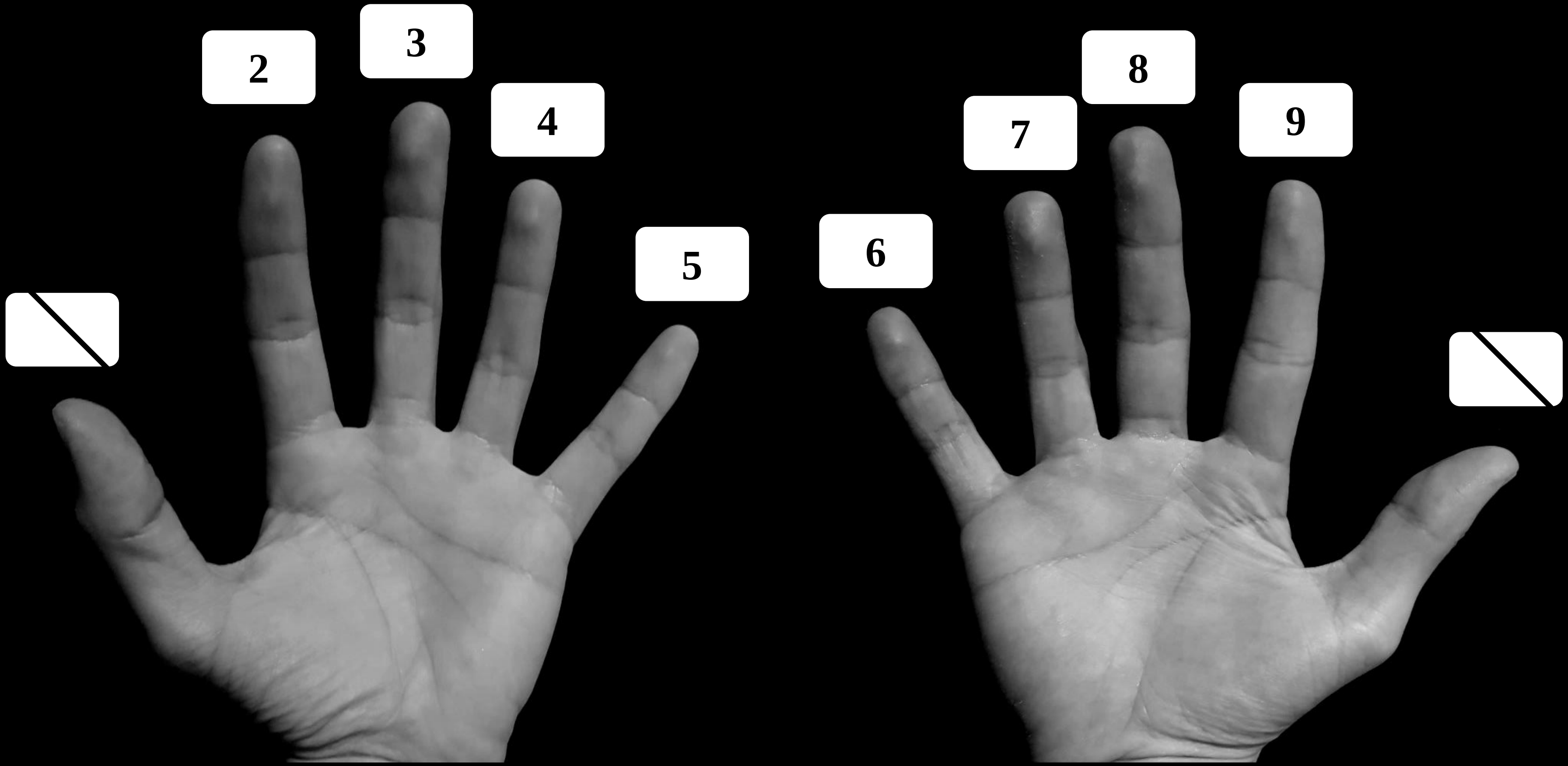

Appendix 2. Unimanual positions

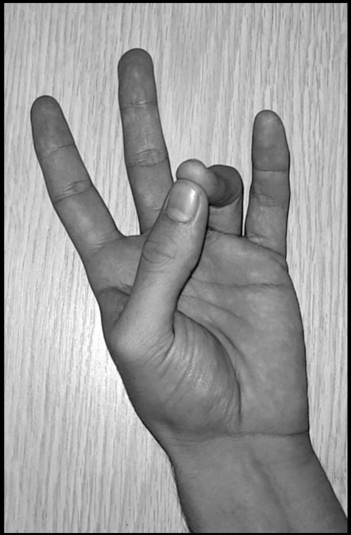

I

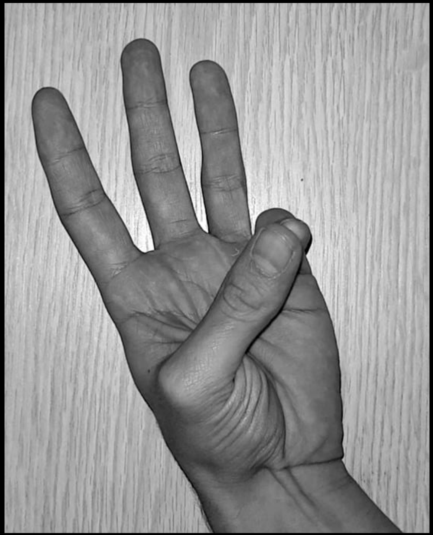

II

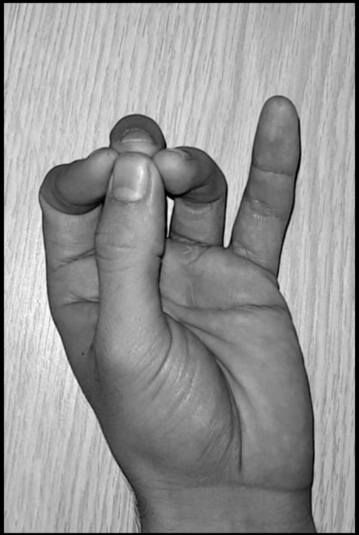

III

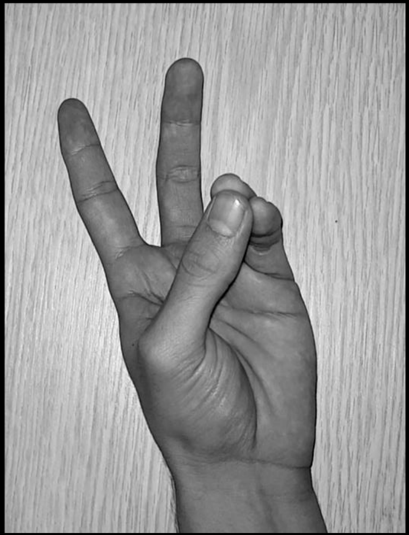

IV

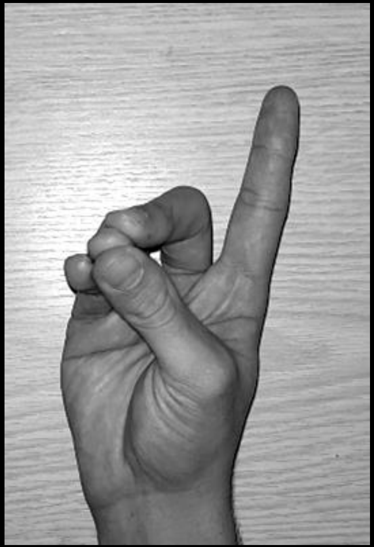

V

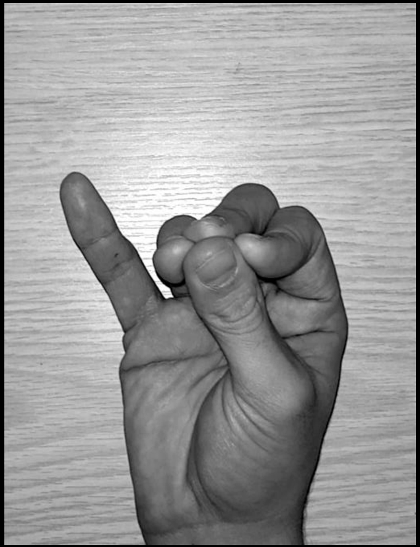

VI

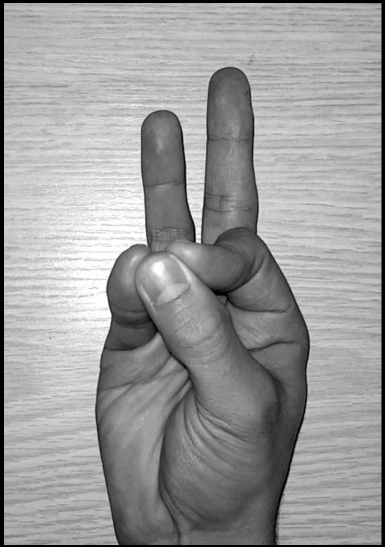

VII

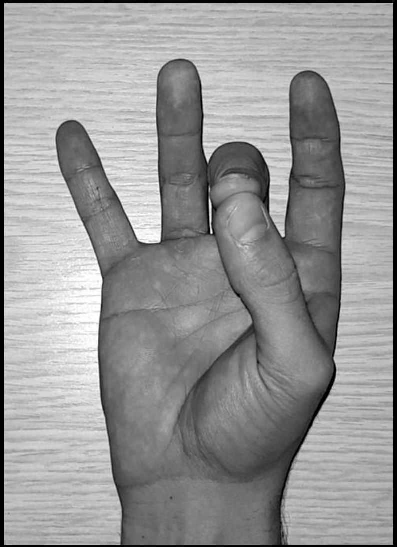

VIII

Appendix 3. Bimanual positions

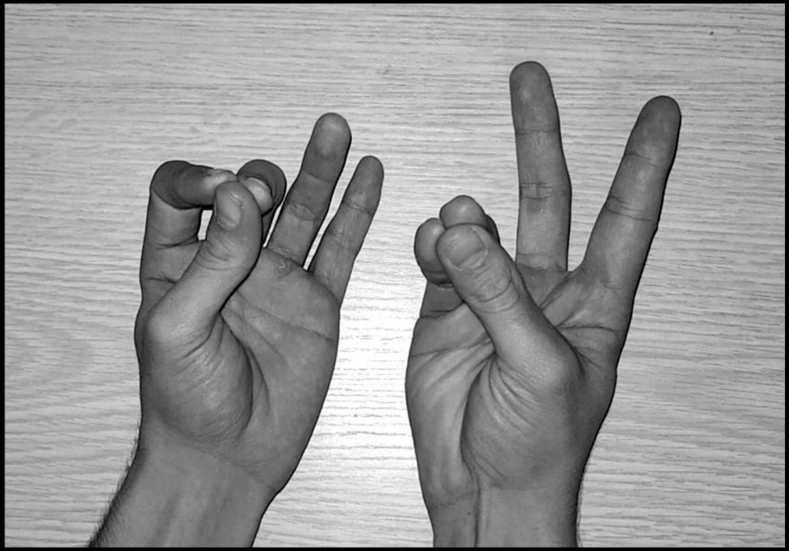

IX

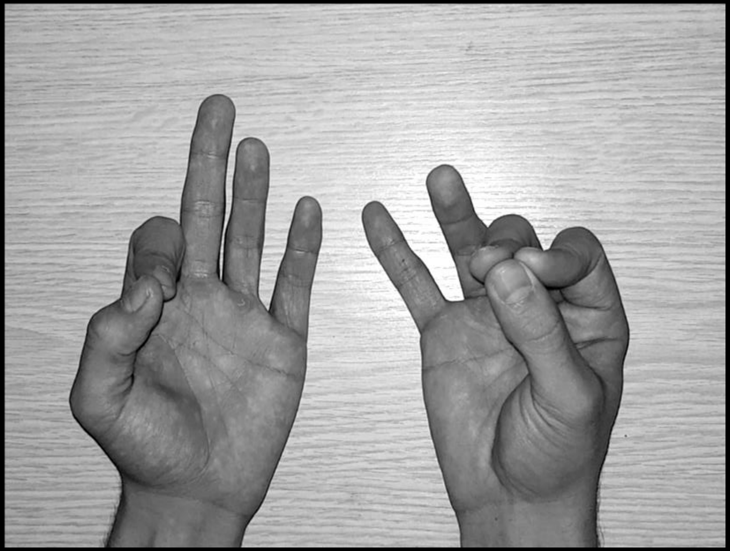

X

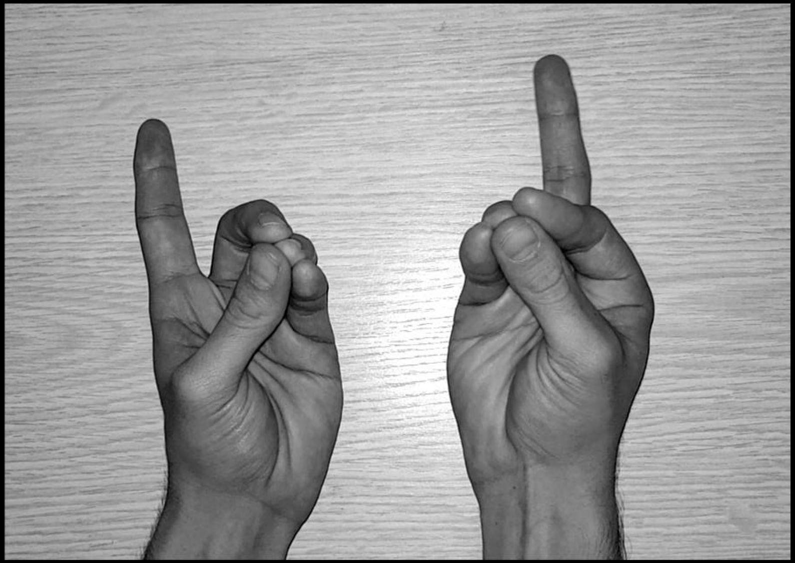

XI

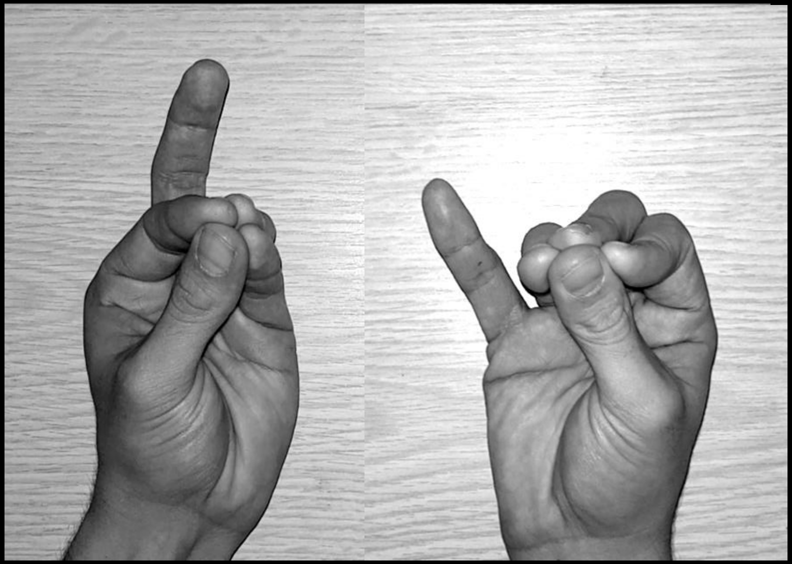

XII
